# Leadership and tempo perturbation affect coordination in medium-sized groups

**DOI:** 10.1038/s41598-021-81504-0

**Published:** 2021-03-02

**Authors:** Bahar Tunçgenç, Eoin Travers, Merle T. Fairhurst

**Affiliations:** 1 School of Psychology, University of Nottingham, Nottingham, UK; 2 Institute of Cognitive and Evolutionary Anthropology, University of Oxford, Oxford, UK; 3 Institute of Cognitive Neuroscience, University College London, London, UK; 4 Faculty of Human Sciences, Institute of Psychology, Bundeswehr University, Munich, Germany; 5 Faculty of Philosophy of Mind, Munich Centre for Neuroscience, LMU, Munich, Germany

**Keywords:** Human behaviour, Social neuroscience

## Abstract

In marching bands, sports, dance and virtually all human group behaviour, we coordinate our actions with others. Coordinating actions in time and space can act as a social glue, facilitating bonding among people. However, much of our understanding about coordination dynamics is based on research into dyadic interactions. Little is known about the nature of the sensorimotor underpinnings and social bonding outcomes of coordination in medium-sized groups—the type of groups, in which most everyday teamwork takes place. In this study, we explored how the presence of a leader and an unexpected perturbation influence coordination and cohesion in a naturalistic setting. In groups of seven, participants were instructed to walk in time to an auditory pacing signal. We found that the presence of a reliable leader enhanced coordination with the target tempo, which was disrupted when the leader abruptly changed their movement tempo. This effect was not observed on coordination with the group members. Moreover, participants’ perceptions of being a follower and group cooperativeness increased in the presence of a leader. This study extends our knowledge about coordination beyond previous work on dyads. We discuss our results in light of sensorimotor coupling and social cohesion theories of coordination in groups.

## Introduction

From marching in a band or a protest^1^ to singing and swaying bodies as part of a ritual^2^, a unifying aspect of human group activities is that they typically rely on temporal coordination. This “muscular bonding”^1^ is thought to act as a “social glue” and facilitate social exchange among individuals^3,4^. Despite mounting interdisciplinary research spanning the anthropological, psychological and neuro-cognitive sciences, much of our knowledge about how we adapt and respond to others’ movements has been based on the study of interactions between either the many (i.e. very large groups^5–8^) or the few (i.e. dyads^9,10^). As such, the underlying mechanisms and consequences of coordination are still poorly elucidated when it comes to medium-sized groups (5–10 individuals), which constitute most of the socially bonded and efficiently performing teams in real-world settings^11–15^. To contribute to filling the gap of how medium-sized groups coordinate, we have taken an interdisciplinary approach and conducted an ecologically valid experiment in a science public engagement event at the *Tate Modern* in London. Using a group walking task, this study shows that when groups of strangers are asked to move in time to a given rhythm, people quickly start stepping in time to this external tempo. Coordination with the target tempo is improved when there is a leader in the group, and is disrupted by a perturbation of the leader’s movement tempo. Further probing group dynamics, we also demonstrate how the presence of a leader influenced people’s feelings of connectedness with the other group members.

Previous work looking at medium-sized groups has been either more observational in nature, describing the consequences of coordinating in groups^16^ or focussed on the sensorimotor dynamics between interacting individuals^9,17–21^. Research on the sensorimotor dynamics of group coordination has provided powerful tools for objectively and dynamically quantifying the degree of coupling between interacting individuals. For example, Richardson et al.^21^ provided one of the first behavioural methods for capturing dynamic group coordination in their study testing groups of six naïve students in rocking chairs. Soczawa-stroncyzk et al.^22^ have shown that interpersonal coordination during a walking task is weaker in groups than in dyads, which may be related to the participants’ receiving sensory information from multiple sources (i.e., other group members) simultaneously. Alderisio et al.^11^ manipulated information exchange through instructed changes in visual coupling (gaze direction) to measure topological changes between two groups of seven individuals waving arms. Similarly, by quantifying network topology, Müller and colleagues have shown how professional choirs coordinated, but also reorganised into smaller groups, depending on whether they were singing canons or in unison^17^. Still within a musical context, work by Wing et al.^20^ have examined the precise temporal adaptation mechanisms at work in musical performances by two professional string quartets. Notably, most of the existing research on the sensorimotor dynamics of medium-sized groups has been constrained to settings in which trained professionals (e.g., surgeons, dancers, musicians or athletes) are observed^23–28^. To our knowledge, the present study is the first to simultaneously test group dynamics both in terms of objective temporal coordination and its consequences on perceived cohesion in a medium-sized group of non-experts.

### Mechanisms underlying interpersonal movement coordination

Coordinating actions with others requires integrating visual and auditory input from others’ actions into one’s own motor system—a process called sensorimotor coupling^29^. Computational modelling and empirical data both support the notion that sensorimotor coupling can emerge spontaneously^30^, as is evident from people’s spontaneous tendency to fall into synchrony with each other while walking^31^, swaying bodies^32^ or moving objects^33^. This tendency to move synchronously with others seems to be so powerful that pairs of people coordinated their movements even in physically challenging contexts, for example, when their rocking chairs had different weights attached to them, making it harder to move in time to the same rhythm^34^.

Such automatic coupling between two independent systems (i.e., in this case, humans) has been explained by coupled oscillatory dynamics^35^. Similar to two oscillators attached together, over time, people start to move in in-phase synchrony with each other and maintain this stable state, where they match the timing and position of their movements. In the absence of a physical string, increased sensory information about the partner’s movements leads to improved coordination among people, for example when the partners had haptic information through holding hands < sup > 31 < /sup >  < sup > 31 < /sup >  < sup > 31 < /sup >  < sup > 31 < /sup > or visual information through attending to each other^23,31,36^ < sup > 23 < /sup >  < sup > 23 < /sup >  < sup > 23 < /sup >  < sup > 23 < /sup > . In daily life settings requiring coordination, there may often be more than one signal in the environment for the individuals to couple with. This introduces the so-called integration-segregation challenge, whereby individuals need to identify the most informative signal to selectively integrate those external rhythms to their own movement and segregate themselves from the external rhythms when they prove inefficient for the purpose^37^.

The strength of coupling and coordination success also depends on who we interact with (e.g. make-up of the group, presence of a leader) and how we interact with them (i.e. nature of the information exchange). Using a joint finger tapping task in dyads, Konvalinka et al.^38^ manipulated the direction and symmetry of information exchange and showed that interacting individuals must both predict and adapt to one another for successful coordination. Further demonstrating the importance of swift adaptation to changing circumstances, it has been shown that in an improvisation game, players go in and out of synchrony following a perturbation^39^ and, adaptation success varies as a function of expertise^40^. The literature on whether having a leader facilitates or hinders coordination is mixed. For instance, a two-person electroencephalography (EEG) study has shown how a leadership profile emerges when one person focuses more on planning and producing a reliable example for a follower to follow, and how this behaviour has a frontally centred neural correlate^41^. Using functional MRI (fMRI), Fairhurst et al.^42^ found similar activity in the inferior frontal gyrus that correlated with a higher propensity to take the lead in maintaining a reliable tempo. On the other hand, research using mirror-game paradigm has shown that coordination improves when dyads engage in joint improvisation as compared to following or being a leader^43^, especially when the dyads are expert actors and musicians^40^.

The factor of temporal reliability and the specific effect of having a leader is less well studied in movement coordination in groups. Vast literature examining the dynamics of coordinated action (but not necessarily movement) problems shows that having good, reliable leaders^44,45^, and enhanced communication^45,46^ among group members can facilitate the coordination of the group members’ actions, even in large groups, though large group size may limit coordination through incurring excessive cognitive demands^46^. One example has demonstrated that although string quartets are able to coordinate precisely without an external leader, greater coupling is observed between the other players and the first violinist^20,47^. Other research has shown that the person in the leader position can employ different timekeeping strategies depending on group size and the wealth of coordination cues available^48^. A major advantage of these temporal coordination studies is the dynamic nature of the interactive tasks, which allow for a change in information exchange and coupling across time. Though typically studied under laboratory conditions, examining dynamic coordination changes has the potential to closely capture the richness of real-world interactions, in which both internal and external conditions may alter coupling between agents.

Neuroimaging research has provided insight into the basis of sensorimotor coupling and interpersonal coordination. For example, several studies using fMRI and EEG have shown that performing matched and coordinated, rather than uncoordinated, actions in dyads is associated with so-called mirror neuron activity^49–51^. Hence, through facilitating action understanding and prediction, mirror neuron system activity may underlie the mirroring aspects of interpersonal coordination. Yet, successful coordination requires not just mirroring others’ actions, but also performing complementary actions that can be swiftly adapted to changing circumstances^52^. The method of hyperscanning has shown that increased interbrain synchronisation is observed when individuals perform a joint task in coordination with each other as compared to when they perform a solo task or coordinate with a computer (see reviews:^53–55^). Dumas et al.^56^ have shown that movement coordination between people with designated roles as models versus imitators was correlated with interbrain synchrony in the alpha-mu band over the right centroparietal regions, which are known to play a key role in top-down modulation and social interactions^57^. Both neurological and behavioural examination of how interactional factors can dynamically change coordination patterns will crucially elucidate how low-level, physical coordination can pave the way for higher-level social cognition and bonding.

### Consequences of interpersonal movement coordination

Coordinating our actions with others in contexts such as a musical ensemble has an obvious positive consequence: it increases everybody’s chances of accomplishing the joint goal. Perhaps more strikingly, however, humans coordinate actions in seemingly goal-less contexts as well, such as when participants in a ritual sway bodies in concert with each other^2^ or when interactants unconsciously start mimicking each other’s postures, gestures and mannerisms^58^. In the past decades, there have been two separate lines of research into the consequences both of interpersonal coordination and of unconscious mimicry.

Research on the social effects of interpersonal movement coordination has revealed that after coordinating movements, people felt more affiliated and similar with each other, and were more likely to help, share and trust their partners^59–63^. In addition to these enhanced feelings of social bonding from a self-oriented perspective, several studies also showed an increase in positive group-oriented feelings. For instance, it has been found that when asked to perform a task in synchrony with others in small groups, people^62–64^ reported feeling more merged or ‘as one’ with the rest of the group as assessed using the Inclusion of Other in Self (IoS) scale^65^. Similarly, when asked to view or listen to videos and audios of individuals waving hands^66^ or walking^67^ or of mother-infant interactions^68^, the observers judged the individuals more as a unit if the actions were performed in a more synchronous, coordinated manner. This growing literature strongly indicates that performing coordinated movements in dyads or small groups facilitates feelings of connectedness both at the individual and at the group level. Yet, more research is needed to understand how changes in temporal coordination is linked to changes in social effects.

Research on the social effects of unconscious mimicry of others’ postures, gestures and mannerisms similarly shows enhanced feelings of connectedness and pro-sociality. Prior research shows that dyads who mimic and are mimicked by others during a conversation experience increased emotional contagion, help and share more of their resources with their partner^69–72^. Moreover, having an explicit motivation or reason to affiliate with a prospective partner increases people’s likelihood of unconsciously mimicking their actions^73–76^. These findings show that on an individual-level, mimicry facilitates social bonding. We also know that both mimickers and mimickees in dyadic interactions report feeling more merged or ‘as one’ with their partners, perceive the interaction to be smoother, and engage in more interdependent, rather than independent, self-construal^70,77^. Yet, how mimicry impacts group-level bonding is unclear, as, to the best of our knowledge, there is no study to date that has examined mimicry in contexts beyond dyads.

### Current study

**Figure 1:**
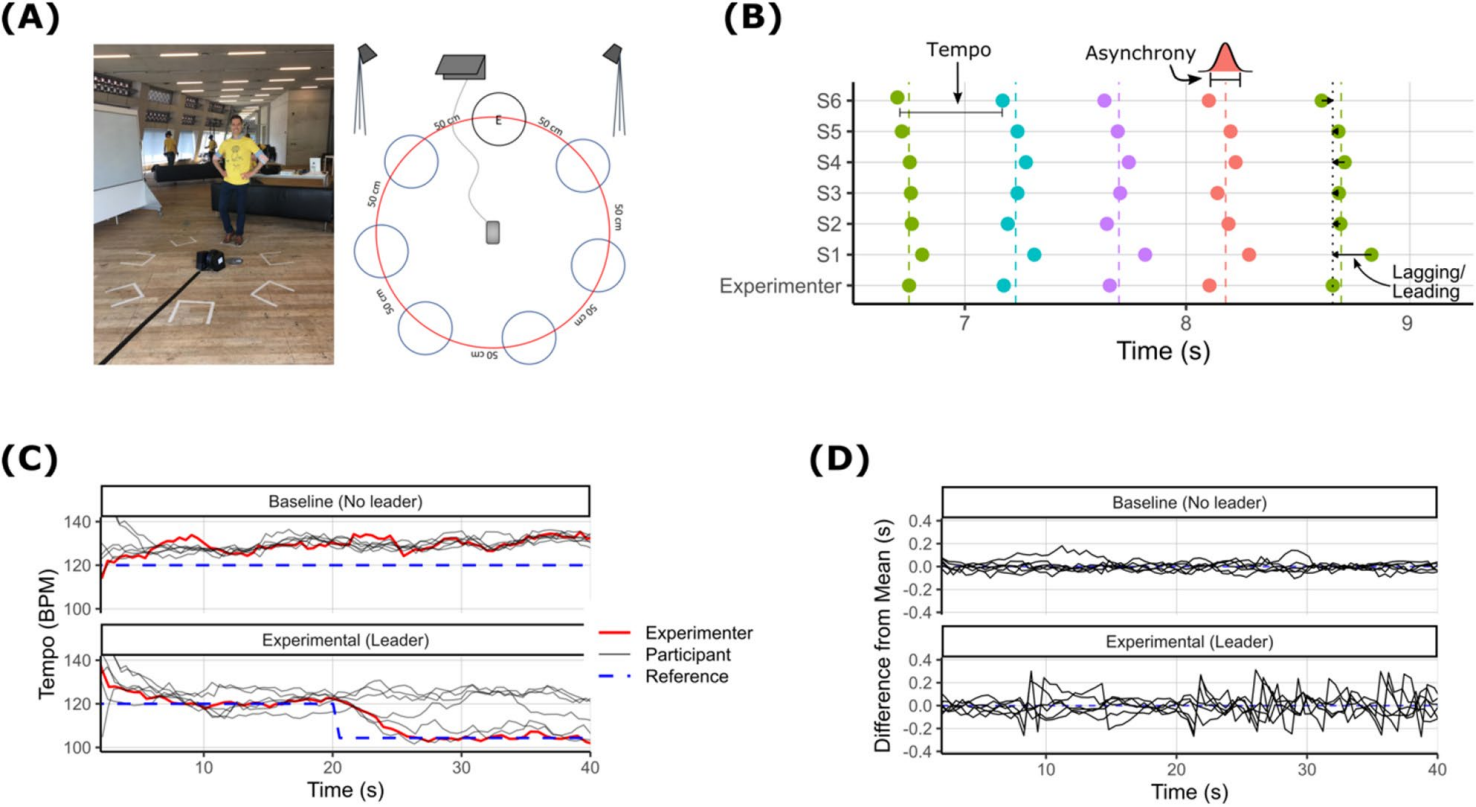
Study design and sample data illustrating dynamic changes in coordination. The R software^78^ package tidyverse version 1.3.0^79^ (https://doi.org/10.21105/joss.01686) was used to plot these figures. (**A**) A novel, and ecological group walking paradigm, where six participants and an experimenter, marked as “E”, formed a circle facing each other around a set of speakers. From the speakers, six initiation beats were heard, after which participants were instructed to continue walking on the spot at the given tempo. (**B**) Clustered step times for each participant and the experimenter over a short segment of a single trial. Dots show the times of individual footsteps. Colour-coding indicates step cluster labels. Coloured dash lines show mean step times. Participant tempo (60/ISI, where ISI is the inter-step interval) is used for calculating coordination with target tempo, asynchrony (standard deviation each group member’s step times) is used for calculating coordination with the group members, and lagging/leading behind/ahead of the experimenter is used for calculating our exploratory measure of coordination with the experimenter. (**C**) Coordination with target tempo for a single group over time. After 20 s in the experimental trial, the experimenter increases their tempo to 104 BPM. For illustration, tempo is calculated in a four-step rolling window for each participant. (**D**) Coordination with the group for the same participants over time. Group coordination is measured as the standard deviation of the six participants’ step times in each step cluster. When group coordination is low (baseline trial), each participant's step times are close to the mean. When coordination is high (experimental trial), step times are widely spread around the mean.

In this study, we explored how, during a brief (40-s) walking task, sensorimotor coupling would enable seven strangers to dynamically become a coordinated group over time. In groups of seven, participants were asked to walk in time to some initiation beats that were briefly presented at the start of the trials (see Fig. 1A). We created conditions in which sensorimotor coupling among members would be high versus low by (a) presenting the group with a reliable leader (in reality, an experimenter), who had access to the movement rhythm of the whole group, and (b) introducing a perturbation in the movement rhythm half-way through the trial by shifting the leader’s movement tempo. Comparing these conditions to a baseline trial in which no leader was introduced, we assessed how well participants coordinated with the initiation beats and with each other (see Fig. 1B–D). We also examined associations between coordination and the participants’ propensity to mimic the experimenter’s gestures and mannerisms. Finally, we investigated the consequences of coordination by examining the group members’ feelings of connectedness.

## Methods

### Participants

The experiment was conducted at the Tate Modern, London, United Kingdom as part of the 2013 *Moving Humans* exhibition. Participants were recruited upon entering the gallery. Over the course of two consecutive testing days, 162 members of the public agreed to participate. A total of 138 individuals (78 women, 59 men, 1 non-binary, ages 16–48, M_age_ = 32.39), making up 23 groups were included in the analysis. Four groups were excluded from analysis: three groups included child participants younger than 16 years of age, and one group had missing movement data due to experimenter error.

All participants were briefed fully on the details of the experiment verbally during recruitment and debriefed after the study was over. Having read through the experiment instructions, participants provided written informed consent. For participants younger than 18 years of age, signed informed consent was obtained from their parents/legal guardians prior to participation. Ethics approval for this study was obtained from the Research Ethics Committee of the School of Advanced Study, University of London. All methods were performed in compliance to the ethical guidelines and regulations. Those whose identifying information is available in the images published provided informed consent for the open-access publication of their image.

### Design

This study had two independent variables: organisation type (no leader baseline trial vs. experimental trial with leader) and perturbation (before vs. after perturbation). Perturbation was applied only in the experimental trial by shifting the experimenter’s movement tempo. As our dependent variables, we assessed: (1) coordination, (2) mimicry behaviour, and (3) feelings of connectedness to the group.

### Procedure

Each session comprised of two trials (i.e., baseline trial and the experimental trial with leader), in which six volunteer participants and an experimenter walked on the spot as a group. The experimenter pretended to be a naïve group member, oblivious to the study aims. Participants and the experimenter made a circle by standing on designated spots that were equidistant from each other. Outside of the circle, there were two cameras that captured the participants’ movements, and a loudspeaker, from which the initial pacing beats were presented.

At the start of each trial, participants heard six initiation beats (120 beats per minute: BPM/500 ms ISI) from the loudspeakers and were instructed to start walking on the third beat. After the six initiation beats, the auditory stimulus ceased, and the participants were instructed to “try their best to keep walking at the same tempo until the trial ends”. There were no explicit instructions to coordinate with the others in the circle, thereby creating a need for each participant to decide whether to follow someone else in the group or their internal rhythm (*a la* integration vs. segregation account mentioned earlier). At the end of the baseline trial, the participants filled out a brief questionnaire and asked to “answer the questions based on their experience of the movement trial and how they felt about their group”. The details of the questions can be found in the Measures section below.

Next, the experimental trial (with leader) started, with the experimenter being introduced to the rest of the group as having been randomly chosen to wear a pair of headphones, from which they would “keep hearing the beats even after the six initiation beats have ceased”. All group members were given the same instructions as in the baseline trial to “try their best to keep walking at the same tempo until the trial ends”. Unbeknownst to the participants, the beats that the experimenter heard changed half-way through the trial to a slower tempo; we assessed how coordination would be disrupted when there is an unexpected change in sensory information. At the end of the experimental trial, the participants filled out the same questionnaire based on their experience of this trial. The participants were thanked and debriefed at the end of the study.

## Measures

### Coordination

Participants performed two group walking tasks, one in the baseline trial (i.e., no leader) and one in the experimental trial (i.e., with leader). Each group walking task lasted for 40 s and started with six initiation beats (120 BPM/500 ms) presented to the participants via loudspeakers. In the experimental trial, the experimenter kept hearing the beats from headphones throughout the trial. To implement the perturbation effect in the experimental trial, the first 20 s comprised of 120 BPM, and the next 20 s comprised of a slower tempo of 104 BPM/575 ms.

The study videos (recorded at 25 frames per second) were coded post-hoc, using the open-source E-LAN software version 5.7^80^ to assess coordination (video refresh rate: 60 Hz). On the E-LAN software, the time points corresponding to step onsets were marked for all group members. The step onset was defined as the first point of contact of the foot with the ground. To enable partial blindness, the experimenter was always coded last, and was blocked from view while the participants’ steps were being coded for. To assess inter-rater reliability, an intraclass correlation coefficient analysis was conducted on the inter-step intervals as judged by two raters, who both coded 30% of the videos. Perfect reliability was reached between the two raters (ICC(1) = 0.99 for both trials).

We used the Mean Shift algorithm^81^, implemented in the Scikit-Learn package for python^82^, to cluster individual step times into coherent “beats” (see Supplementary Figure S1). This clustering approach was necessary as participants did not reliably begin on the same beat, and occasionally paused or missed steps. The bandwidth parameter was manually tuned to 0.25 s, which produced satisfactory results. We excluded 3884 beats (0.82% of the total) with more than 7 footsteps, indicating that a participant had stepped more than one step in a single beat. We calculated individual participants’ movement tempo by finding the interval in seconds between successive steps (inter-step interval, ISI), excluding cases where a beat was missed, and converted this to tempo in beats per minute: BPM = 60/ISI.

We used two metrics of coordination: (1) coordination with the target tempo and (2) coordination with the group members. To measure coordination with the target tempo, the target tempo in each section of the experiment (104 BPM/575 ms in the second half of the experimental trial, 120 BPM/500 ms otherwise) was subtracted from each participants’ tempo to obtain the tempo error. To measure coordination with the group members, we calculated the standard deviation of the step times of six participants in a group within each beat to estimate group-level asynchrony. Finally, in an exploratory attempt to assess leader–follower relationships, on each beat, we subtracted the experimenter’s step time from that of the participants’ to obtain a measure of how far before (*leading*, a negative difference) or after (*lagging*, a positive difference) the experimenter they stepped. Tempo error and the lagging/leading index were averaged within each group to yield group-level estimates.

### Mimicry behaviour

To examine spontaneous mimicry of gestures, the experimenter scratched their arms and legs, and touched their faces during the walking task eight times per trial. Through post-hoc coding of the study videos using E-LAN, we determined how many times each participant performed the target actions (i.e., arm and leg scratching, face touching) after the experimenter had performed at least one such action within each trial. Two raters coded 30% of the videos for the frequency of mimicry observed; a Pearson correlation analysis between the two raters’ scores revealed perfect inter-rater reliability (*r*(82) = 0.96, *p* < 0.0001). As participants were very rarely coded as having mimicked more than once per trial (5% of cases), our analyses focus on whether or not participants were coded as mimicking at all. The analysis of mimicry was conducted at participant-level.

### Self-report measures of feelings of connectedness

After the walking phase of each trial, participants completed a questionnaire. The first questions on the questionnaire included basic demographic information about the participants’ age, gender, and years of music and dance experience, if any. This was followed by five items related to the participants’ feelings about the group. On continuous scales with marked end-points, participants were asked: (Q1) how much they felt like a leader versus a follower during the experience, (Q2) how small or large they felt during the experience, (Q3) how much the other group members seemed cooperative or competitive during the experience, and (Q4) how much they would like to do another activity with the same group members in the future. The participants’ answers to Questions 1–4 were normalised to fit a scale ranging from 0 to 10. The final item (Q5) assessed how merged the participants felt with their group using a 6-point version of the IoS scale^65^. The analysis of these self-report measures was conducted at participant-level.

### Statistical analyses

For group-level analyses, participant-level measures (tempo error, lagging/leading, mimicry, self-report measures) were averaged within each group (group asynchrony, defined as the standard deviation of step times on each beat, is already a group-level measure). We present each of our coordination measures both as a function of time on each trial and averaged within trial halves (the first and second 20 s of each trial). For inference, we conduct 2 (trial: baseline or experimental) × 2 (time: first or second half) ANCOVAs, averaging over steps (n ≈ 40 per trial half) and over participants (n = 6 per group). Mean participant age, gender, years of dance experience, and years of music experience were included as covariates, but all results were unchanged when these terms where omitted. Unexpected interactions with the covariates are noted in the main text and explored in more detail in Supplementary Materials. Tests were conducted using the afex package^83^ for R version 1.3.959^78^. Post-hoc contrasts were conducted using the emmeans package^84^, with an significance threshold Bonferroni-adjusted for two comparisons to α < 0.025.

Analyses of mimicry behaviour and self-report outcomes were conducted at the participant-level. We fit a logistic mixed model to analyse whether or not participants mimicked the experimenter, and a linear mixed model to analyse the self-report scales. We included random intercepts for each group, using the lme4^85^ and lmerTest^86^ packages for R. These models allow us to examine participant-level predictors while taking into account the nesting of individual participants within groups.

Satterthwaite degrees of freedom were used for linear models. To compare baseline and experimental trials, we fit models with main effects of trial. To explore factors that predict these outcomes in experimental trials, we included the following predictors: average coordination with the target tempo, coordination with the group members, leading/lagging scores, difference scores for each of these measures (second half minus first half, capturing sensitivity of these measures to perturbation), and our standard covariates (age, gender, music and dance experience).

## Results

### Coordination

**Figure 2:**
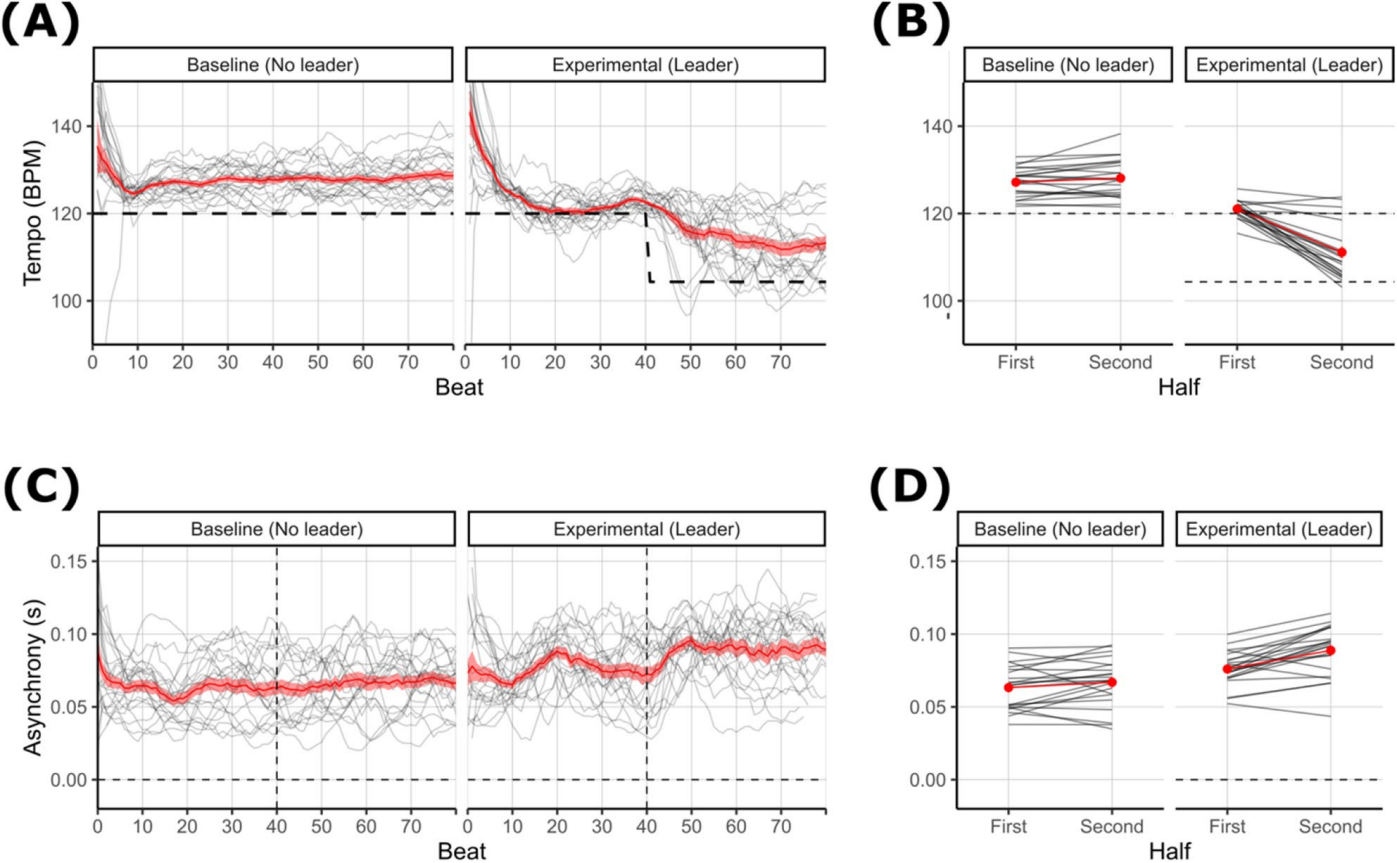
Coordination measures over time (**A**–**C**) and aggregated within each half of each trial (**B**–**D**). Black lines show individual groups. Red lines show mean ± SEM. Temporal data is smoothed using a 6-beat sliding window for visualisation. The R software^78^ package tidyverse version 1.3.0^79^ (https://doi.org/10.21105/joss.01686) was used to plot these figures. (**A**,**B**) Coordination with the target tempo over time (**A**) and aggregated across groups (**B**). The y-axis shows movement tempo in beats per minute (BPM) and dashed lines represent the target tempo, which changes from 120 to 104 BPM after 20 s on experimental trials. (**C**,**D**) Coordination with the group members over time (**C**) and aggregated across groups (**D**). The y-axis shows the standard deviation of the step times of six participants in a group, in seconds.

Coordination with the target tempo and the group members are shown in Fig. 2. Qualitatively, participants’ movements were too fast in the baseline trial, but were closer to the target tempo in the first half of the experimental trial. This may indicate the benefit of following the experimenter and/or a learning effect over time. Participants failed to fully adjust their tempi to match the faster speed set by the experimenter in the second half of the experimental trial. Closer inspection reveals that most groups did in fact increase their tempo to match the experimenter, while a few did not do so at all. To examine these patterns statistically, we conducted 2 (trial: baseline vs experimental) × 2 (time: first half vs second half) ANCOVA tests on the metric of coordination with the target tempo and the metric of coordination with the group members.

Coordination with the target tempo, shown in Fig. 2A,B, is assessed by a 2 × 2 ANCOVA. Significant main effects of trial, *F*(1,18) = 101.24, η*p*^2^ = 0.69,* p* < 0.001, and time, *F*(1,18) = 45.67, η*p*^2^ = 0.25, *p* < 0.001, were qualified by a significant trial × time interaction, *F*(1,18) = 78.67, η*p*^2^ = 0.33,* p* < 0.001. There was an unpredicted three-way interaction of years of music education × trial × time, *F*(1,18) = 5.25,* η*_*p*_^2^ = 0.03,* p* = 0.034, such that groups with more years of music education were better able to adjust their tempo to match the experimenter in the second half of the experimental trial (see Supplementary Materials). Post-hoc contrasts showed that tempo did not change throughout the baseline trial, *t*(35.8) = 1.028, *p* = 0.622, but significantly decreased between the first and second halves of the experimental trial, *t*(35.8) = 6.323,* p* < 0.001, and that tempo was significantly reduced in the first half of the experimental trial compared to the first half of the baseline, *t*(27.5) = 4.705,* p* < 0.001. In both halves of both trials, participants’ movements were significantly faster than the target tempos, *t*(21) > 2.504,* p* < 0.022.

Coordination with the group members, shown in Fig. 2C,D, is assessed by a 2 × 2 ANCOVA. We found a significant main effect of trial, *F*(1,18) = 23.95, *η*_*p*_^2^ = 0.29, *p* < 0.0001, indicating greater asynchrony on experimental trials than baseline trials, and a significant main effect of time, *F*(1,18) = 15.15, *η*_*p*_^2^ = 0.08, *p* = 0.001, indicating greater asynchrony in the second half than the first. However, these effects were qualified by a significant trial × time interaction, *F*(1,18) = 9.56,* η*_*p*_^2^ = 0.03,* p* = 0.006. Post-hoc contrasts showed a significant effect of time on the experimental trial, *t*(32.2) = 4.961,* p* < 0.001, but not on the baseline trial, *t*(32.2) = 1.421,* p* = 0.165. An additional post-hoc test showed that asynchrony was significantly greater on the first half of the experimental trials than on the second half of baseline trials, although this effect did not survive correction for multiple comparisons, *t*(21) = 2.118, *p* = 0.046. We found an unpredicted age × trial interaction, *F*(1,18) = 5.40,* η*_*p*_^2^ = 0.08,* p* = 0.032, such that younger groups were better coordinated than older groups on baseline trials but did not differ on experimental trials (see Supplementary Materials).

Finally, we explored how much participants led or lagged behind the experimenter in a 2 × 2 ANCOVA. Participants significantly lead the experimenter (stepped before the experimenter did on each beat) in the first half of the experimental trials only, *t*(21) = −5.234, *p* < 0.001, all other *t* < 1.64, *p* > 0.117. Full ANCOVA results are reported in Supplementary Materials.

### Mimicry

Participants were coded as having mimicked the experimenter at least once on 17% of baseline trials (SD = 16%, SEM = 3%) and 23% of experimental trials (SD = 22%, SEM = 5%). This difference was not statistically significant, *b* = 0.44,* z* = 1.318, *p* = 0.187*.* In our analysis of the experimental trials, none of the behavioural measures significantly predicted mimicry (see Supplementary Materials). Mimicry was, however, more common in participants with more years of dance education, *b* = 1.54,* z* = 2.011,* p* = 0.044, and female participants, *b* = 1.65,* z* = 2.454, *p* = 0.014*.*

### Self-report measures

For each of our five self-report measures, Table 1A reports the mean ratings on baseline and experimental trials, along with the estimated difference between the trials and the corresponding significance level. Table 1B,C reports regression weights for the behavioural and demographic predictors of self-report ratings on experimental trials, estimated by fitting a multiple regression model for each self-report item. Full regression tables can be found in Supplementary Materials.

**Table 1:** Self-report measures across trials and as predicted by other measures. (**A**) Mean (± SD) self-report ratings from Baseline and Experimental trials, and estimated differences between each trial type. (**B**,**C**) Regression weights for behavioural and demographic predictors of self-report ratings on experimental trials. Weights are estimated by fitting a multiple regression model for each self-report item.

	Self-report items				
	Felt following	Felt large	Felt competitive	Would do again	Self-other merging
**(A) Baseline versus experimental trials**					
Baseline	4.36 (2.25)	4.95 (1.93)	3.75 (2.46)	6.40 (2.37)	3.37 (1.23)
Experimental	6.69 (2.71)	4.72 (2.15)	2.77 (2.23)	6.27 (2.55)	3.30 (1.34)
Estimated difference	2.71***	− 0.19	− 1.15***	− 0.18	− 0.06
**(B) Behavioural predictors**					
Mean asynchrony	− 9.05	− 15.57	1.52	20.9	17.76
δ Asynchrony	1.59	4.54	− 3.87	− 63.27*	6.07
Mean Tempo	− 0.18*	0.01	0.07	− 0.1	0.03
δ Tempo	− 0.12*	0.08	0.01	− 0.02	0.02
Mean lag	− 4.46	0.62	− 1.31	4.99	− 2.89
δ Lag	0.63	− 2.28	0.98	− 0.34	− 0.08
**(C) Demographic predictors**					
Dance edu	− 0.25	1.29**	0.38	− 0.81	0.22
Music edu	0.36	− 0.04	− 0.09	− 0.13	− 0.38
Age	0.01	0.00	− 0.02	0.02	0.00
Female	− 0.48	− 1*	− 0.22	1.12*	0.26

*p < .05; **p < .01, ***p < .001, uncorrected*.*

## Discussion

Coordinating within medium-sized groups is an essential aspect of human social behaviour. Yet, extant literature on the causes and consequences of interpersonal coordination has largely focused on dyadic interactions, which cannot address group dynamics that are essential to bonding, coordination and cooperation^10^. This study explored how seven strangers would dynamically evolve into a coordinated group over a relatively short amount of time in a novel and ecological group walking task. We manipulated the degree of sensorimotor coupling among group members by introducing a reliable leader, and by applying an unexpected perturbation by shifting the leader’s movement tempo. We showed that through increased sensorimotor coupling in the experimental trial (with leader), participants coordinated with the target tempo better, which subsequently was disrupted when the leader changed their movement tempo. Having a leader and perturbation did not affect coordination with other group members, suggesting that in this brief task, which required participants to maintain the external beats they heard at the start of the trials, participants chose to follow these external rhythms rather than following other group members who were going in and out of synchrony with the target tempo. These findings expand our current knowledge about coordination in medium-sized groups by demonstrating its causes and consequences, and the dynamic changes, in an ecologically-valid setting.

Investigating dyads and very large groups is not conducive for a full understanding of how interpersonal coordination takes place in real-world settings, due either to the absence of group dynamics or to the difficulty with examining individual characteristics. Analyses of large databases show that more stable^12^ and socially bonded^14^ interactions develop in medium-sized groups. For instance, research suggests that medium-sized teams of around 5–10 individuals tend to accomplish complex tasks better and have higher coordination and cohesion in the cases of most businesses^13^, medical surgical teams^87^, sports teams^15^, and musical ensembles^11^. One reason why medium-sized groups function more optimally than dyads or large groups might have to do with the rich information exchange^14^. Yet, this rich information exchange may come with a cost, as, unlike in dyads, in groups, simultaneous sensorimotor coupling with many other individuals may be cognitively and neurally demanding^22,46^. Hence, especially with complex tasks, performing coordinated movements may reduce the cognitive load through activating the mirror neuron system, and help facilitate collaboration as a group^88^. As such, it is possible that for small- to medium-sized groups in the workplace, on the street, at the park or in a ritual, it is easier to coordinate at a muscular level very quickly, and without specific training, which may scaffold into being able to coordinate at other, higher socio-cognitive levels.

In the present study, we implemented a basic coordination task that mimics something we do every day; departing from tasks involving simple limb coordination such as hand waving^11^ or finger tapping^89^, our group walking task was chosen for its enactive and ecological qualities by involving the whole body (“muscular bonding”; see: McNeill^1^; Wiltermuth and Heath^59^). Crucially, this design allows us to track how individuals shift between the instructed task of maintaining a given tempo and coupling their movements to the other walkers in the circle. Typically, a distinction is made between spontaneous and intentional coordination^90^. However, here we suggest that a further layer of complexity may exist in either case, such that an individual coordinating with a group of others can shift attention from the external timekeeper (e.g., the pacing signal) to specific members of the group and back, as either source of information becomes more informative in the process of the coordinated action (for similar suggestions, also see:^22,37^). Indeed, what we are measuring in this paradigm is how participants’ movements are affected by the social pull versus the external rhythmic stimuli. Our results show that in this brief walking task of 40 s, participants chose to follow the task instructions and follow the target tempo. Future research can examine how the coordination dynamics would change in longer movement trials. This highly flexible design also provides a tool for examining how costs of coordinating in groups are either increased (e.g., by introducing a perturbation) or mitigated (e.g., presence of a reliable leader) by improved information exchange.

Mounting research has shown that creating conditions of enhanced sensorimotor coupling increases coordination between partners. Enhancement of sensorimotor coupling has been attained by having people hold hands or see each other^11,31,36^. Another implicit way of enhancing sensorimotor coupling, often used as a way to create synchronous and asynchronous conditions in experimental studies, is to explicitly instruct the participants to follow another person^34,91^. Similarly, the few existing studies conducted in medium-sized group settings have shown the importance of sensorimotor coupling for successful movement coordination^23,25^. Prior research on expert orchestra musicians show that having a leader in the group provides a reference point for all other members and thereby improves coordination in tasks that involve high skill and complexity^24,92^. Here, we further this work in a real-world group setting with non-expert, naïve individuals who were strangers to each other. As expected, we found that coordination with the target tempo was higher in the presence of a reliable leader compared to the condition with no leader. The presence of a leader may have yielded better coordination through enhancing sensorimotor coupling and/or increasing the group members’ perception of shared goals and joint action. Further, we found that coordination was disrupted when the leader abruptly changed their movement tempo. This finding adds to our understanding of how coordination takes place in groups by quantifying not only its emergence (i.e., coupling) but also its disruption (i.e., uncoupling) when task complexity and sensorimotor coupling demands increase.

Bridging the sensorimotor coupling mechanisms of coordination with its social consequences, we also examined how coordination would be associated with^93–95^ mimicry and feelings of closeness. We did not find any effects of having a leader or coordination on mimicry, which may have been due to very small amount of mimicry observed. Previous studies examining coordination in small groups of 3–4 people indicate that group coordination increases cooperation, feelings of connectedness^62,63^ and group members’ understanding of others’ mental states^96^. Our findings extend this knowledge by showing enhanced feelings of connectedness with the group in the experimental trial, in which participants’ coordination with the external tempo was higher than at baseline. Closer analysis of the individual questionnaire items suggests that while feelings of being a follower and perceiving others as being cooperative increased, participants were not more willing to engage in a future interaction with the same people and did not report feeling as one or merged with the group. The finding of perceiving others as more cooperative may indicate that the presence of a leader had a higher-level impact on shared goals and joint action. While it is possible that due to the increased neural cost of coordination, social bonding effects observed in dyads may be reduced in medium-sized groups, it may also be that the brevity of our task (40 s) and the unfamiliarity of the group members may have caused the observed patterns. Given the lack of precedent on the consequences of coordination in medium-sized groups, more research is needed before drawing firm conclusions from our self-report findings.

This study had several restrictions. Firstly, the length of the sessions was restricted, which prevented us from including a no-leader condition after the experimental trial. This meant that the experimental trial was compared only with the baseline trial that preceded it, which led us to observe an order effect in the metric of coordination with the other group members. Future research should implement fully counterbalanced designs to circumvent this issue. Moreover, due to the limited facilities available, we were not able to obtain motion tracking data from our participants. Instead, we video-recorded the participants’ movements and computed two types of variability-based coordination metrics. Importantly, these purpose-designed metrics satisfy the key considerations in quantifying aspects of group coordination in a comprehensive manner^21^ when time-series data is not available to conduct phase-based analysis. Future studies could combine motion tracking data to provide richer quantifiable data about the specific phase relations among the participants’ movements. Finally, it will be important for future research to examine whether the found effects on feelings of cooperativeness of the group members would indeed translate into participants acting more cooperatively in task-based measures.

For a richer theoretical account of coordination dynamics and how they shape social behaviour in groups, an interdisciplinary approach investigating both the causes and consequences of temporal coordination is essential. Based on our findings, we suggest that such an account needs to include investigations of groups of varying sizes, and interactions between individuals who are not able to rely on expertise or familiarity to coordinate. Finally, we posit that there may be a functional scaling factor that describes how the degree of coordination changes with group size depending on task complexity (considering both cognitive load and social demands) and duration. A one-size-fits-all account is unlikely to describe human group behaviour and social bonding**—**instead, we need to examine dynamic real-world interactions to gain particular insight into how we do things with others and rapidly adapt to changing circumstances.

## Supplementary Information

Supplementary Information.
