# Supplementary material for: Leadership and tempo perturbation affect coordination in medium-sized groups
Source: Sci Rep. 2021 Mar 2;11:4940. doi: 10.1038/s41598-021-81504-0 (PMC7925598; doi:10.1038/s41598-021-81504-0)
Supplement: Supplementary file 1 — Supplementary Information. [file 41598_2021_81504_MOESM1_ESM.docx]

**Supplementary Materials
for
Leadership and tempo perturbation affect coordination in medium-sized groups**

Bahar Tunçgenç^1,2*^, Eoin Travers^3^, Merle T. Fairhurst^4,5^

^1^School of Psychology, University of Nottingham, Nottingham, UK;
^2^ Institute of Cognitive and Evolutionary Anthropology, University of Oxford, Oxford, UK;
^3^Institute of Cognitive Neuroscience, University College London, UK;
^4^Institute of Psychology, Faculty of Human Sciences, Bundeswehr University, Munich, Germany;
^5^Munich Centre for Neuroscience and Faculty of Philosophy of Mind, LMU, Munich, Germany

**Supplementary Methods**

**Automatic Clustering of Step Times**

Participants did not always step in perfect synchrony. Instead, participants within a group would commonly start at different times or skip steps before establishing a stable rhythm. As a result, it is not feasible group step times within each group in an ordinal way, as the 10^th^ step for one participant could be at the same time as the 8^th^ step for another, and the 12^th^ step of a third, despite these participants being otherwise synchronised. This issue is illustrated in Figure S1.

To address this, we used the Mean Shift algorithm, implemented in the Scikit-Learn package for python, to group individual steps into temporal clusters. To do this, we pooled all step times across all participants within a single trial, and used the Mean Shift algorithm, with bandwidth=0.25 s, to detect clusters in the data in an unsupervised way. The bandwidth parameter was set by hand, but is consistent with the fact that the average inter-step interval was close to 0.5 s.

The clustering algorithm does not make use of information about which participant produced each step, and so occasionally identified clusters with more than one step per participant. We therefore excluded 3,884 beats (0.82% of the total) with more than 7 footsteps.


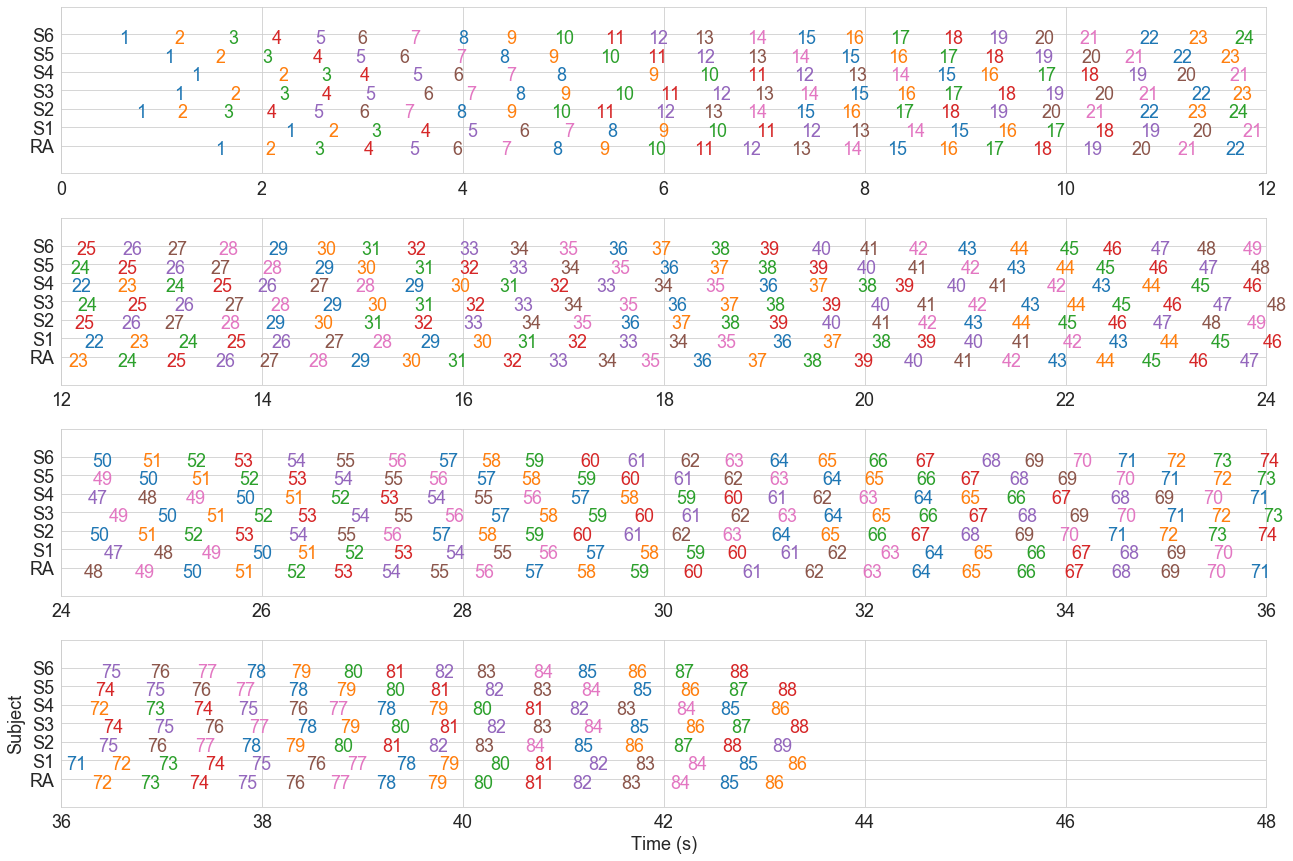


**Figure S1.** Step times for a single group (six subjects and the experimenter: RA) across one trial of 44 seconds. Steps for each person are shown along a horizontal line, with time represented on the x-axis. Numbers and colour-coding denote the ordinal labels, that is, the 1^st^, 2^nd^, 3^rd^ step, etc., for each person.


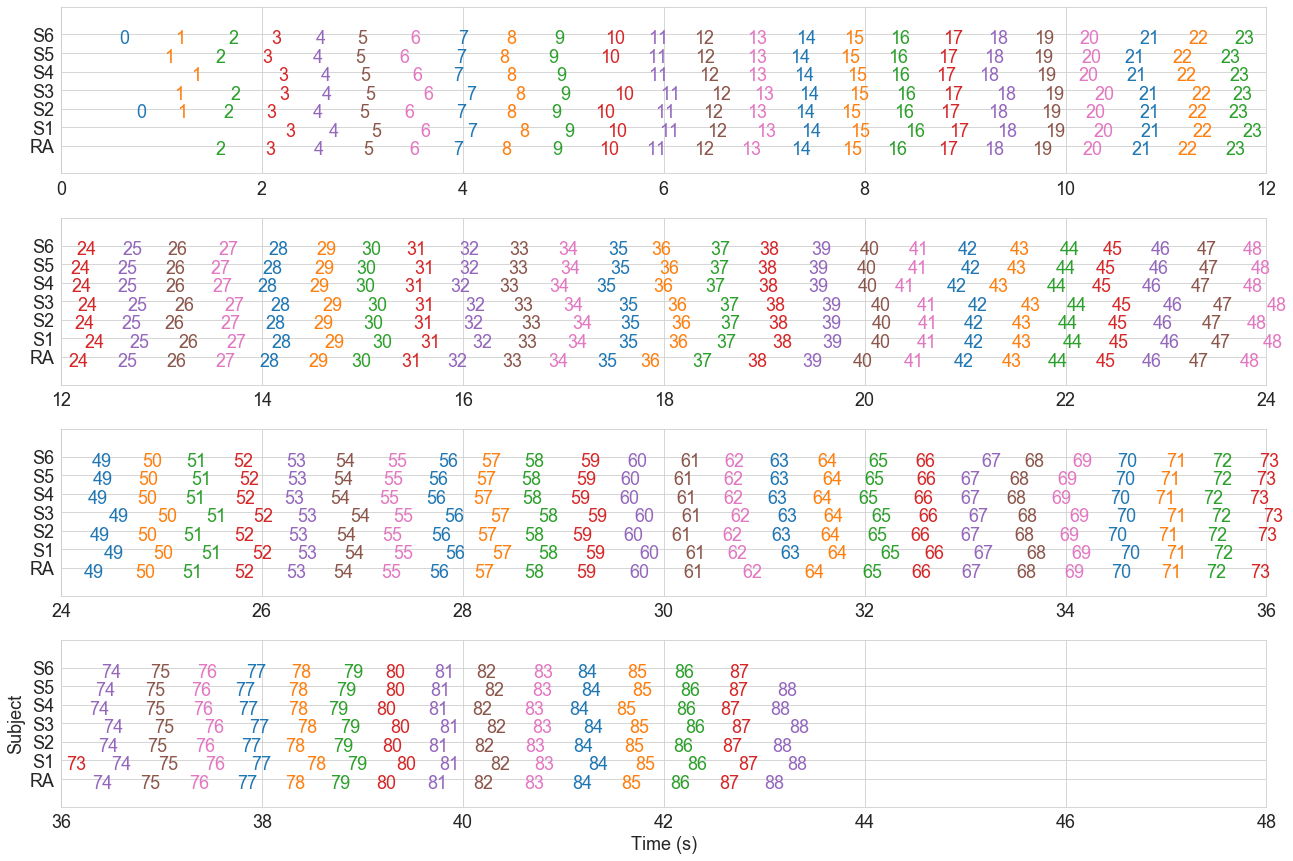


**Figure S2.** Step times for the same group, clustered using the Mean Shift algorithm. Numbers and colour-coding here denote the cluster into which each step has been classified.

**Supplementary Results**

1. **Coordination**

| *Table S1. ANCOVA table for analysis of average tempo.* | | | | | | | |
| --- | --- | --- | --- | --- | --- | --- | --- |
| Family | Term | F | DF1 | DF2 | MSE | p | ges |
| Predictors | Trial | 101.24 | 1 | 18 | 29.01 | < .001 | .692 |
|  | Half | 45.67 | 1 | 18 | 9.76 | < .001 | .254 |
|  | Trial × Half | 78.67 | 1 | 18 | 8.25 | < .001 | .332 |
| Covariates | Age mean | 1.47 | 1 | 18 | 25.57 | .242 | .028 |
|  | Years dance mean | 1.78 | 1 | 18 | 25.57 | .198 | .034 |
|  | Years music mean | 0.74 | 1 | 18 | 25.57 | .400 | .014 |
| Predictor × Covariate interactions | Age × Trial | 0.02 | 1 | 18 | 29.01 | .890 | .000 |
|  | Age × Half | 0.52 | 1 | 18 | 9.76 | .481 | .004 |
|  | Years dance × Trial | 0.10 | 1 | 18 | 29.01 | .757 | .002 |
|  | Years dance × Half | 1.69 | 1 | 18 | 9.76 | .210 | .012 |
|  | Years music × Trial | 1.04 | 1 | 18 | 29.01 | .321 | .023 |
|  | Years music × Half | 0.35 | 1 | 18 | 9.76 | .563 | .003 |
|  | Age × Trial × Half | 0.48 | 1 | 18 | 8.25 | .496 | .003 |
|  | Years dance × Trial × Half | 1.31 | 1 | 18 | 8.25 | .267 | .008 |
|  | Years music × Trial × Half | 5.25 | 1 | 18 | 8.25 | .034 | .032 |

| *Table S2. ANCOVA table for analysis of group asynchrony (SD of step times).* | | | | | | | |
| --- | --- | --- | --- | --- | --- | --- | --- |
| Family | Term | F | DF1 | DF2 | MSE | p | ges |
| Predictors | Trial | 23.95 | 1 | 18 | 0.00 | < .001 | .288 |
|  | Half | 15.15 | 1 | 18 | 0.00 | .001 | .083 |
|  | Trial × Half | 9.56 | 1 | 18 | 0.00 | .006 | .027 |
| Covariates | Age mean | 2.04 | 1 | 18 | 0.00 | .170 | .057 |
|  | Years dance mean | 0.03 | 1 | 18 | 0.00 | .862 | .001 |
|  | Years music mean | 0.95 | 1 | 18 | 0.00 | .343 | .027 |
| Predictor × Covariate interactions | Age × Trial | 5.52 | 1 | 18 | 0.00 | .030 | .085 |
|  | Age × Half | 0.37 | 1 | 18 | 0.00 | .548 | .002 |
|  | Years dance × Trial | 1.63 | 1 | 18 | 0.00 | .218 | .027 |
|  | Years dance × Half | 0.09 | 1 | 18 | 0.00 | .761 | .001 |
|  | Years music × Trial | 0.24 | 1 | 18 | 0.00 | .632 | .004 |
|  | Years music × Half | 0.25 | 1 | 18 | 0.00 | .620 | .002 |
|  | Age × Trial × Half | 0.09 | 1 | 18 | 0.00 | .773 | .000 |
|  | Years dance × Trial × Half | 0.12 | 1 | 18 | 0.00 | .731 | .000 |
|  | Years music × Trial × Half | 0.62 | 1 | 18 | 0.00 | .443 | .002 |

| *Table S3. ANCOVA table for analysis of average lagging/leading behind/ahead of the experimenter.* | | | | | | | |
| --- | --- | --- | --- | --- | --- | --- | --- |
| Family | Term | F | DF1 | DF2 | MSE | p | ges |
| Predictors | Trial | 23.40 | 1 | 18 | 0.00 | < .001 | .292 |
|  | Half | 10.66 | 1 | 18 | 0.00 | .004 | .069 |
|  | Trial × Half | 16.25 | 1 | 18 | 0.00 | .001 | .110 |
| Covariates | Age mean | 8.17 | 1 | 18 | 0.00 | .010 | .160 |
|  | Years dance mean | 6.16 | 1 | 18 | 0.00 | .023 | .125 |
|  | Years music mean | 1.54 | 1 | 18 | 0.00 | .231 | .035 |
| Predictor × Covariate interactions | Age × Trial | 1.02 | 1 | 18 | 0.00 | .326 | .018 |
|  | Age × Half | 3.06 | 1 | 18 | 0.00 | .098 | .021 |
|  | Years dance × Trial | 0.37 | 1 | 18 | 0.00 | .552 | .006 |
|  | Years dance × Half | 0.13 | 1 | 18 | 0.00 | .725 | .001 |
|  | Years music × Trial | 2.55 | 1 | 18 | 0.00 | .128 | .043 |
|  | Years music × Half | 0.93 | 1 | 18 | 0.00 | .347 | .006 |
|  | Age × Trial × Half | 1.19 | 1 | 18 | 0.00 | .290 | .009 |
|  | Years dance × Trial × Half | 0.40 | 1 | 18 | 0.00 | .537 | .003 |
|  | Years music × Trial × Half | 0.22 | 1 | 18 | 0.00 | .646 | .002 |


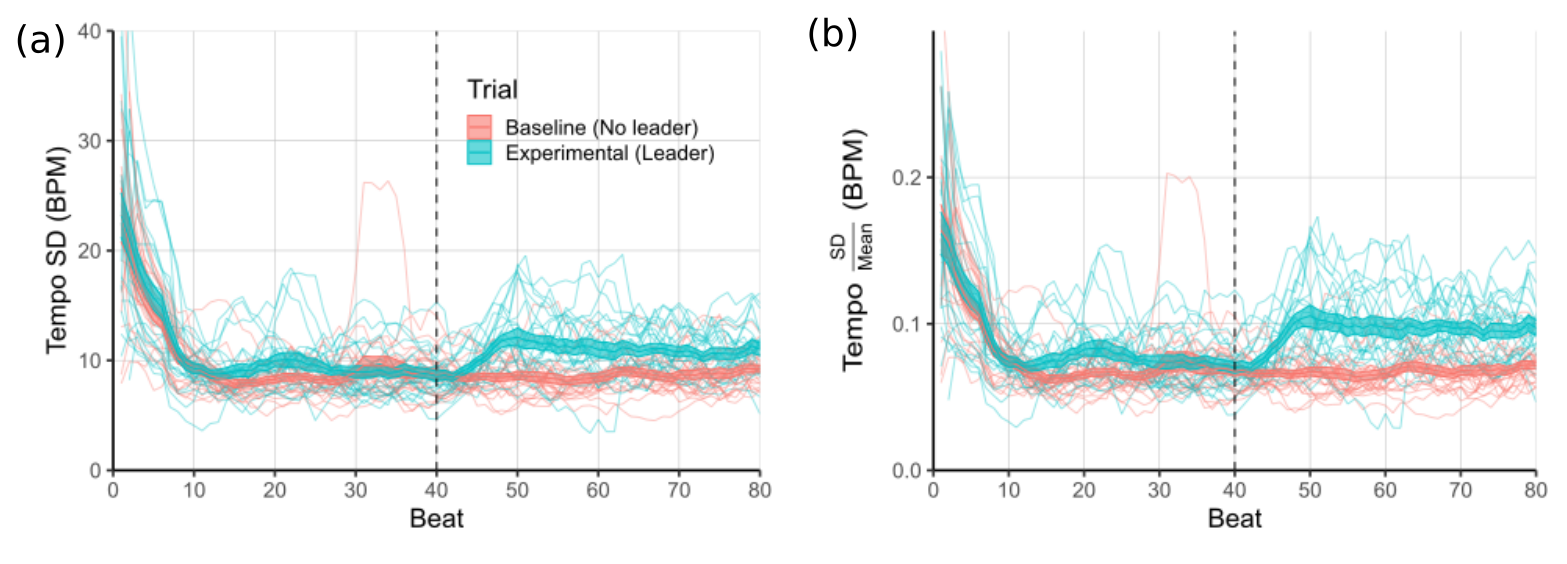


**Figure S3.** **(a)** Standard deviation of step tempo over time within each group.

**(b)** Normalised tempo standard deviation (standard deviation of tempo divided by mean tempo). Tempo variability did not differ between baseline and experimental trials over the first 40 beats of each trial. Variability increased after beat 40 in the experimental trials, where the experimenter increased their step tempo from this time.


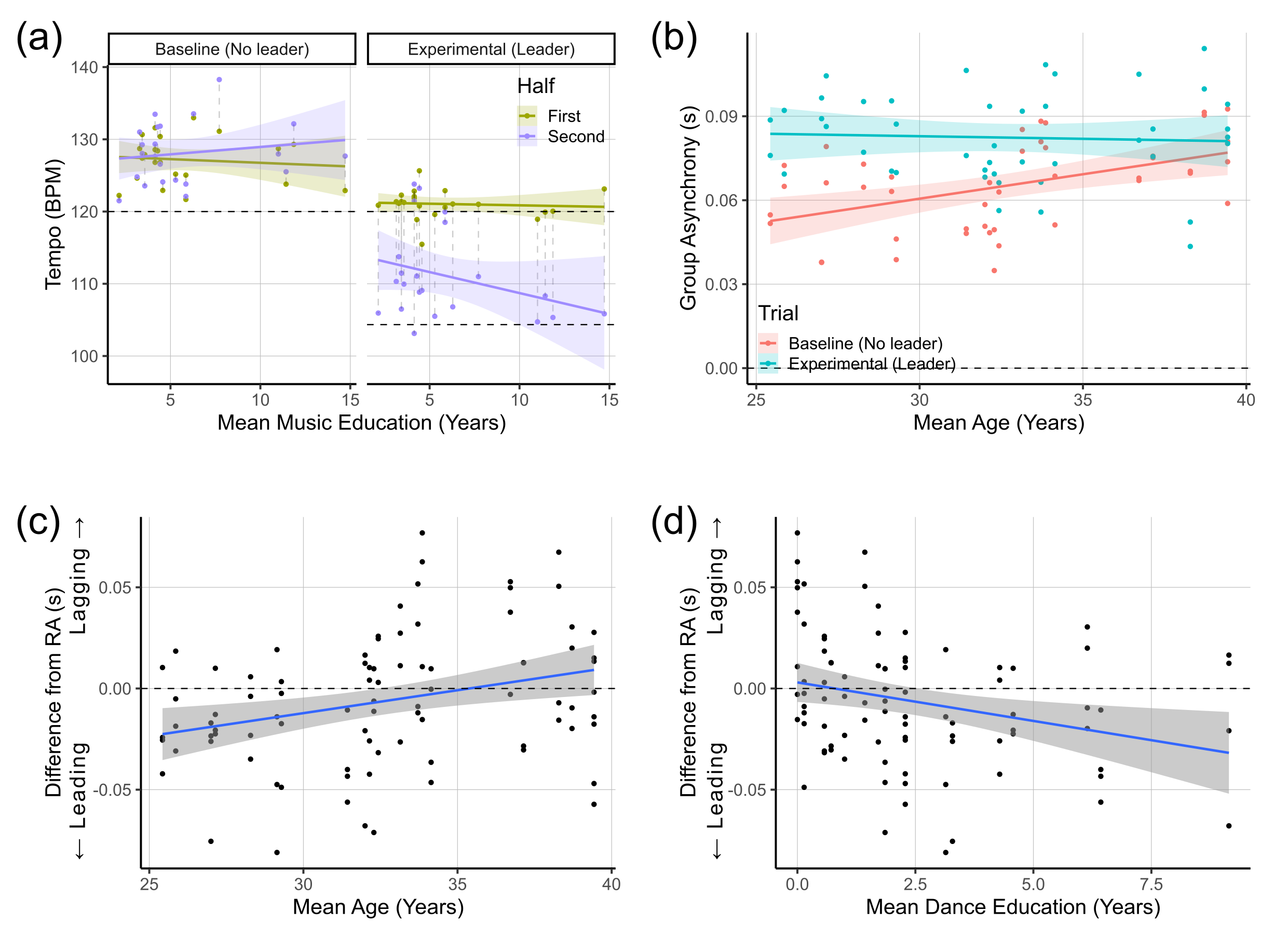


**Figure S4.** Unpredicted interactions with covariates. Error bars show standard error of simple regression.

**(a)** Years of music education × Trial × Half interaction on mean tempo. On the experimental trials, groups with more years of music experience were better able to slow their tempi to match the experimenter in the second half.

**(b)** Age × Trial interaction on group asynchrony. On baseline trials, younger groups had lower asynchrony (were more in time with each other) than older groups.

**(c)** Main effect of Age on lagging. Younger groups tended to step ahead of the experimenter. Older groups tended to step behind.

**(d)** Main effect of Years of dance education on lagging. Groups with more dance experience tended to step ahead of the experimenter.

1. **Mimicry**

| *Table S4. Fixed effects from logistic model comparing the probability of mimicry on baseline and experimental trials.* | | | | |
| --- | --- | --- | --- | --- |
| Term | b | SE | z value | p |
| Intercept | -1.840 | 0.363 | -5.070 | 0.000 |
| Experimental vs. Baseline | 0.439 | 0.333 | 1.318 | 0.187 |

| *Table S5. Fixed effects from model examining predictors of mimicry within experimental trials.* | | | | |
| --- | --- | --- | --- | --- |
| Term | b | SE | z value | p |
| Intercept | -1.779 | 0.469 | -3.794 | 0.000 |
| Mean Asynchrony | 9.166 | 32.075 | 0.286 | 0.775 |
| δ Asynchrony | -40.531 | 36.063 | -1.124 | 0.261 |
| Mean Tempo | 0.104 | 0.085 | 1.223 | 0.221 |
| δ Tempo | -0.014 | 0.072 | -0.195 | 0.845 |
| Mean Lag | -0.248 | 7.016 | -0.035 | 0.972 |
| δ Lag | -3.332 | 6.188 | -0.538 | 0.590 |
| Dance education | 1.538 | 0.765 | 2.011 | 0.044 |
| Music education | 0.328 | 0.589 | 0.558 | 0.577 |
| Age | 0.003 | 0.027 | 0.120 | 0.904 |
| Female | 1.625 | 0.662 | 2.454 | 0.014 |

1. **Self-report measures**

**“Felt Following”**

*Table S6. Fixed effects from model comparing responses to the “Felt following” item on baseline and experimental trials.*

| Term | b | SE | DF | t value | p |
| --- | --- | --- | --- | --- | --- |
| Intercept | 4.202 | 0.244 | 61.507 | 17.214 | < .001 |
| Experimental vs. Baseline | 2.714 | 0.323 | 236.127 | 8.407 | < .001 |

| *Table S7. Fixed effects from model examining predictors of the “Felt following” item within experimental trials.* | | | | | |
| --- | --- | --- | --- | --- | --- |
| Term | b | SE | DF | t value | p |
| Intercept | 27.277 | 8.632 | 118 | 3.160 | .002 |
| Mean Asynchrony | -9.050 | 19.870 | 118 | -0.455 | .650 |
| δ Asynchrony | 1.595 | 22.495 | 118 | 0.071 | .944 |
| Mean Tempo | -0.176 | 0.071 | 118 | -2.473 | .015 |
| δ Tempo | -0.120 | 0.051 | 118 | -2.358 | .020 |
| Mean Lag | -4.460 | 5.410 | 118 | -0.824 | .411 |
| δ Lag | 0.629 | 4.675 | 118 | 0.134 | .893 |
| Dance education | -0.250 | 0.533 | 118 | -0.469 | .640 |
| Music education | 0.360 | 0.468 | 118 | 0.768 | .444 |
| Age | 0.013 | 0.021 | 118 | 0.628 | .531 |
| Female | -0.477 | 0.471 | 118 | -1.014 | .313 |

**“Felt Large”**

| *Table S8. Fixed effects from model comparing responses to the “Felt large” item on baseline and experimental trials.* | | | | | |
| --- | --- | --- | --- | --- | --- |
| Term | b | SE | DF | t value | p |
| Intercept | 4.852 | 0.191 | 257 | 25.462 | < .001 |
| Experimental vs. Baseline | -0.191 | 0.270 | 257 | -0.708 | .480 |

| *Table S9. Fixed effects from model examining predictors of the “Felt large” item within experimental trials.* | | | | | |
| --- | --- | --- | --- | --- | --- |
| Term | b | SE | DF | t value | p |
| Intercept | 5.753 | 7.902 | 118 | 0.728 | .468 |
| Mean Asynchrony | -15.572 | 18.188 | 118 | -0.856 | .394 |
| δ Asynchrony | 4.538 | 20.590 | 118 | 0.220 | .826 |
| Mean Tempo | 0.008 | 0.065 | 118 | 0.127 | .899 |
| δ Tempo | 0.078 | 0.047 | 118 | 1.666 | .098 |
| Mean Lag | 0.621 | 4.952 | 118 | 0.125 | .900 |
| δ Lag | -2.276 | 4.279 | 118 | -0.532 | .596 |
| Dance education | 1.291 | 0.488 | 118 | 2.646 | .009 |
| Music education | -0.035 | 0.429 | 118 | -0.083 | .934 |
| Age | 0.004 | 0.020 | 118 | 0.226 | .822 |
| Female | -1.002 | 0.431 | 118 | -2.327 | .022 |

**“Felt Competitive”**

| *Table S10. Fixed effects from model comparing responses to the “Felt competitive” item on baseline and experimental trials.* | | | | | |
| --- | --- | --- | --- | --- | --- |
| Term | b | SE | DF | t value | p |
| Intercept | 3.597 | 0.256 | 42.344 | 14.026 | < .001 |
| Experimental vs. Baseline | -1.148 | 0.283 | 236.183 | -4.055 | < .001 |

| *Table S11. Fixed effects from model examining predictors of the “Felt competitive” item within experimental trials.* | | | | | |
| --- | --- | --- | --- | --- | --- |
| Term | b | SE | DF | t value | p |
| Intercept | -4.997 | 8.008 | 112.491 | -0.624 | .534 |
| Mean Asynchrony | 1.524 | 23.115 | 18.593 | 0.066 | .948 |
| δ Asynchrony | -3.875 | 26.122 | 18.789 | -0.148 | .884 |
| Mean Tempo | 0.067 | 0.065 | 116.214 | 1.021 | .310 |
| δ Tempo | 0.007 | 0.047 | 113.869 | 0.140 | .889 |
| Mean Lag | -1.306 | 4.959 | 116.874 | -0.263 | .793 |
| δ Lag | 0.982 | 4.342 | 113.845 | 0.226 | .821 |
| Dance education | 0.379 | 0.474 | 115.982 | 0.801 | .425 |
| Music education | -0.087 | 0.413 | 114.281 | -0.210 | .834 |
| Age | -0.017 | 0.019 | 117.407 | -0.888 | .377 |
| Female | -0.217 | 0.418 | 115.784 | -0.520 | .604 |

**“Would Do Again”**

| *Table S12. Fixed effects from model comparing responses to the “Would do again” item on baseline and experimental trials.* | | | | | |
| --- | --- | --- | --- | --- | --- |
| Term | b | SE | DF | t value | p |
| Intercept | 6.599 | 0.288 | 39.171 | 22.906 | < .001 |
| Experimental vs. Baseline | -0.184 | 0.303 | 236.060 | -0.608 | .544 |

| *Table S13. Fixed effects from model examining predictors of the “Would do again” item within experimental trials.* | | | | | |
| --- | --- | --- | --- | --- | --- |
| Term | b | SE | DF | t value | p |
| Intercept | 16.101 | 9.357 | 103.779 | 1.721 | .088 |
| Mean Asynchrony | 20.901 | 23.304 | 19.412 | 0.897 | .381 |
| δ Asynchrony | -63.273 | 26.364 | 19.630 | -2.400 | .026 |
| Mean Tempo | -0.097 | 0.077 | 107.605 | -1.261 | .210 |
| δ Tempo | -0.020 | 0.055 | 105.294 | -0.355 | .723 |
| Mean Lag | 4.986 | 5.841 | 109.785 | 0.854 | .395 |
| δ Lag | -0.336 | 5.075 | 102.248 | -0.066 | .947 |
| Dance education | -0.814 | 0.569 | 118.000 | -1.431 | .155 |
| Music education | -0.131 | 0.498 | 117.219 | -0.263 | .793 |
| Age | 0.018 | 0.023 | 117.291 | 0.783 | .435 |
| Female | 1.120 | 0.502 | 117.999 | 2.232 | .027 |

**“Self-Other Merging”**

| *Table S14. Fixed effects from model comparing responses to the “Self-other merging” item on baseline and experimental trials.* | | | | | |
| --- | --- | --- | --- | --- | --- |
| Term | b | SE | DF | t value | p |
| Intercept | 3.308 | 0.145 | 41.497 | 22.862 | < .001 |
| Experimental vs. Baseline | -0.055 | 0.161 | 235.448 | -0.344 | .731 |

| *Table S15. Fixed effects from model examining predictors of the “Self-other merging” item within experimental trials.* | | | | | |
| --- | --- | --- | --- | --- | --- |
| Term | b | SE | DF | t value | p |
| Intercept | -2.060 | 4.956 | 109.290 | -0.416 | .678 |
| Mean Asynchrony | 17.762 | 13.646 | 16.816 | 1.302 | .211 |
| δ Asynchrony | 6.066 | 15.426 | 16.995 | 0.393 | .699 |
| Mean Tempo | 0.031 | 0.041 | 113.768 | 0.765 | .446 |
| δ Tempo | 0.024 | 0.029 | 110.721 | 0.836 | .405 |
| Mean Lag | -2.892 | 3.077 | 115.007 | -0.940 | .349 |
| δ Lag | -0.079 | 2.689 | 110.001 | -0.029 | .977 |
| Dance education | 0.215 | 0.296 | 116.820 | 0.729 | .468 |
| Music education | -0.384 | 0.258 | 114.977 | -1.488 | .140 |
| Age | 0.004 | 0.012 | 117.896 | 0.335 | .738 |
| Female | 0.260 | 0.261 | 116.677 | 0.997 | .321 |
